# Objective Structured Clinical Examinations Provide Valid Clinical Skills Assessment in Emergency Medicine Education

**DOI:** 10.5811/westjem.2014.11.22440

**Published:** 2014-12-12

**Authors:** Joshua Wallenstein, Douglas Ander

**Affiliations:** Emory University, Department of Emergency Medicine, Atlanta, Georgia

## Abstract

**Introduction:**

Evaluation of emergency medicine (EM) learners based on observed performance in the emergency department (ED) is limited by factors such as reproducibility and patient safety. EM educators depend on standardized and reproducible assessments such as the objective structured clinical examination (OSCE). The validity of the OSCE as an evaluation tool in EM education has not been previously studied. The objective was to assess the validity of a novel management-focused OSCE as an evaluation instrument in EM education through demonstration of performance correlation with established assessment methods and case item analysis.

**Methods:**

We conducted a prospective cohort study of fourth-year medical students enrolled in a required EM clerkship. Students enrolled in the clerkship completed a five-station EM OSCE. We used Pearson’s coefficient to correlate OSCE performance with performance in the ED based on completed faculty evaluations. Indices of difficulty and discrimination were computed for each scoring item.

**Results:**

We found a moderate and statistically-significant correlation between OSCE score and ED performance score [r(239) =0.40, p<0.001]. Of the 34 OSCE testing items the mean index of difficulty was 63.0 (SD =23.0) and the mean index of discrimination was 0.52 (SD =0.21).

**Conclusion:**

Student performance on the OSCE correlated with their observed performance in the ED, and indices of difficulty and differentiation demonstrated alignment with published best-practice testing standards. This evidence, along with other attributes of the OSCE, attest to its validity. Our OSCE can be further improved by modifying testing items that performed poorly and by examining and maximizing the inter-rater reliability of our evaluation instrument.

## INTRODUCTION

The unpredictable nature of emergency department (ED) patient encounters limits the standardization of ED-based clinical evaluation, particularly when that evaluation is focused upon defined tasks and competencies, or when it must be completed within a short time period. Emergency medicine (EM) educators typically must perform both comparative assessments of multiple learners as well as progressive evaluation of individual learners. Reproducibility of clinical scenarios and encounters enhances the objectivity of such evaluations. This, however, can be a challenge given the random nature of ED encounters, particularly when the time period for assessment is relatively brief. The provision of safe and high-quality patient care further limits the ability to assess decision-making among novice learners in high-risk situations. To overcome these challenges, EM educators have increasingly turned to additional methods of clinical evaluation that are reproducible, non-threatening to patient safety, and provide standardized assessment of defined skills in specific encounter types. As with all forms of assessment in medical education, these methods must demonstrate evidence of validity to be interpreted in a meaningful manner.

Patient simulation has emerged as one such tool that can evaluate performance in specific encounters and competencies in multiple learners over an extended time period. While the term simulation generally is used in reference to high-fidelity mannequins, the “human” model of simulation obtained through the use of standardized patients (SPs) has emerged as a standard of assessment in undergraduate and graduate medical education. The objective structured clinical examination (OSCE) first introduced in 1975^1^ has become a staple of competency evaluation in medical education^2^ and is also a component of the U.S. medical licensure process. The newly-released EM milestones, part of the Accreditation Council of Graduate Medical Education’s (ACGME) New Accreditation System (NAS), lists the OSCE as a suggested evaluation method in multiple performance areas.^3^

The OSCE and high-fidelity simulation share much in common. They are both able to recreate specific patient-care scenarios for multiple learners and evaluate specific competencies among those learners. They are both reproducible, allowing for standardized evaluation of multiple groups of learners, and for evaluating performance over time in individual learners. While high-fidelity simulation has the added capabilities of simulating and modifying abnormal physical exam findings, the OSCE is superior in evaluating diagnostic skills, such as the history and physical, and in evaluating communication and interpersonal skills. A growing body of literature supports the use of OSCEs and SPs in EM education. EM-based OSCEs have been used to evaluate a diverse range of skills, including advanced communication tasks such as death disclosure^4^ and intimate partner violence counseling.^5^ OSCEs have also been used in EM to evaluate educational interventions by comparing learner performance in intervention and control groups^6^ and to predict future trainee performance among post-graduate trainees.^7^

In 2007 we developed a management-focused OSCE as a tool for clinical assessment of students in our required fourth-year EM clerkship. One of the limitations of the traditional OSCE format is that it is not particularly well suited for evaluating patient management skills or clinical decision-making, both of which are core learning objectives of our clerkship. To better evaluate the acquisition of these skills we made a substantive change to the traditional OSCE format that can best be described as a blending of the traditional SP encounter and the interactive “role-play” style of patient-management typified by the American Board of Emergency Medicine (ABEM) oral certification examination. In our OSCE students interact not only with an SP, but also with a case facilitator who through role-play portrays multiple individuals (patient, family member, resident nurse, consulting physician), and provides additional data (vital sign changes, laboratory and radiographic test results) based on student-initiated management steps. The case facilitator additionally evaluates student performance using a standardized evaluation instrument. SPs and facilitators receive both formal initial training and ongoing evaluation and feedback to maximize the standardization of patient portrayal and student evaluation.

While multiple studies have demonstrated the validity of the OSCE as an assessment method, it has been suggested that the validity of a particular OSCE depends on the *application* of the test, including its accuracy of reflection, scoring measures, and characteristics of the participating subjects.^8^ In that regard, it is important to determine if our unique and non-traditional OSCE format is indeed a valid assessment of clinical skills in EM trainees.

A key component of a test’s validity is evidence of correlation with other established evaluation methods. In both undergraduate and graduate EM training the most established clinical evaluation method is the ED performance evaluation completed by supervising faculty based on a subject’s clinical performance over the course of one or more ED shifts. We hypothesized that student performance on our EM OSCE would correlate with their clinical performance in the ED, as determined by the cumulative evaluation of all “end-of-shift” evaluations completed by faculty and residents. An additional source of a test’s validity is the characteristics of its individual components or items, particularly the indices of difficulty and discrimination. These indices are valuable measures of the “usefulness” of individual testing items in differentiating high and low performers. We further hypothesized that our OSCE test-item analysis would demonstrate adherence to published best-practice guidelines for these measures.

## METHODS

This was a prospective cohort study. We submitted the study to our local institutional review committee, which determined that it met criteria for exemption of further review.

Our study population was comprised of medical students in our institution’s required EM clerkship between September 2009 and February 2011. The OSCE was administered in simulated exam rooms at our institution’s Clinical Skills Center. Clinical evaluation during the clerkship took place at up to five of our affiliated hospitals, which include a tertiary care referral center, an urban county hospital, a mixed academic/community hospital, and two pediatric centers.

An EM clerkship OSCE program was developed under the leadership of the EM clerkship director and the associate director of our center’s clinical skills program who oversees SP recruitment, training, and oversight. Cases were developed by the Department of Emergency Medicine Education Committee and designed to represent the broad spectrum of disease, acuity, and patient demographics that would typically be encountered during our EM clerkship (Table). The cases were further designed to align with the learning objectives specified in a national curriculum guide for EM clerkships.^9^

The OSCE is a required component of our EM clerkship and is administered during the final week of each clerkship block. Performance on the examination constitutes 15% of a student’s final grade. Students receive an orientation to the nature of this OSCE by the clerkship director and the associate director of the clinical skills program. At the start of each case students are provided with a triage report, listing the chief complaint, vital signs, and pertinent demographic and medical history. Students are given 15 minutes to perform patient evaluation and management and reach a disposition. In several of the cases performance of early resuscitative measures is indicated, and students perform these and other management tasks through verbalization of patient care orders to the case facilitator. Pre-scripted updates in vital signs and clinical status are given to the students based on the management steps they perform. Students may request diagnostic tests, such as laboratory and radiographic studies, the results of which are provided in a simulated real-time manner. Each case requires a patient disposition decision by the conclusion of the case.

**Figure 1:**
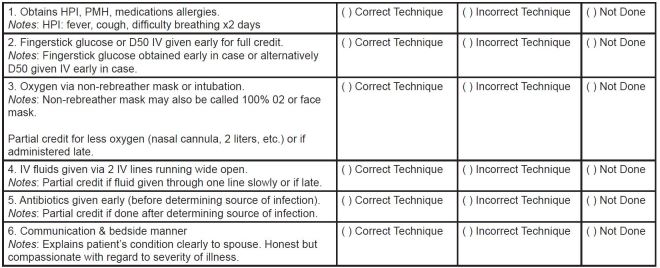
Descriptive example of OSCE evaulation instrument (Altered mental status/sepsis case) *HPI*, history of the previous illness; *PMH*, past medical history; *IV*, intravenous; *OSCE,* objective structured clinical examination

At the core of each of the five cases in our OSCE are pre-selected key historical features and physical exam findings, 3–5 critical actions (including diagnostic and therapeutic tasks), and specific communication objectives (such as giving bad news, discussing advance directives, and obtaining informed consent). Our task-based evaluation instrument is anchored to both quantitative and qualitative assessment of these specific tasks. Performance of the history and physical is scored based upon the number of key features and exam findings elicited. Performance of critical actions is evaluated based upon the number of actions performed, as well as the completeness and timeliness of each task. Communication and interpersonal skills is evaluated based on performance in relation to a specific goal or task. A descriptive example of the evaluation instrument is shown in Figure 1. While we recognize the value of a global rating scale as an assessment tool, we specifically did not include global ratings in our assessment as student performance was assessed by our case facilitators. We felt that they received appropriate training to perform task-based assessment but did not have the background or training to perform a global assessment of performance.

All testing items in each case were weighted equally, and all cases within the OSCE were weighted equally (each case constituted 20% of the final OSCE grade). The ratio of total points earned to total points possible to earn determined a student’s final grade, and was expressed on a 0–100 scale.

We recruited our case facilitators from our institution’s pool of SPs. As our non-physician evaluators are assessing performance of medical tasks, we specifically sought evaluators with a healthcare background. Our cohort of casefacilitators includes retired nurses, paramedics and emergency medical technicians. Regardless of background, all SPs and evaluators complete a formal training program that includes presentation of case goals and objectives, review of case scripts, overview and use of the evaluation instrument, and detailed description of full and partial performance for each critical action. To maintain standardization of patient portrayal and evaluation standards, SPs and evaluators are regularly observed (via remote video feed) by both EM faculty and our clinical skills program leadership. They receive individual feedback on their performance and also participate in regular group conferences.

Students’ clinical performance in the ED is measured using our institution’s clinical evaluation assessment tool which is uniformly used by all clinical clerkships. This tool utilizes a 9 item anchored 1–5 Likert scale to assess competencies related to medical knowledge, clinical practice, procedural skills, and communication, and a 5 item scale to assess professionalism. Based on an equal weighting of all completed faculty and resident evaluations, students receive a final clinical score as well as sub-scores in each competency area.

Using Pearson’s coefficient we assessed the correlation between final OSCE score and final clinical score. Index of difficulty and index of discrimination were computed for each scoring item and compared to best-practice standards.

## RESULTS

We enrolled 278 medical students in the study. Five students did not participate in the OSCE due to unresolvable schedule conflicts, and others were found to have missing or incorrect data. Complete data from all five cases was available for 239 students. All students received a final ED clinical score representing an equal weighting of all completed shift evaluation forms.

**Figure 2:**
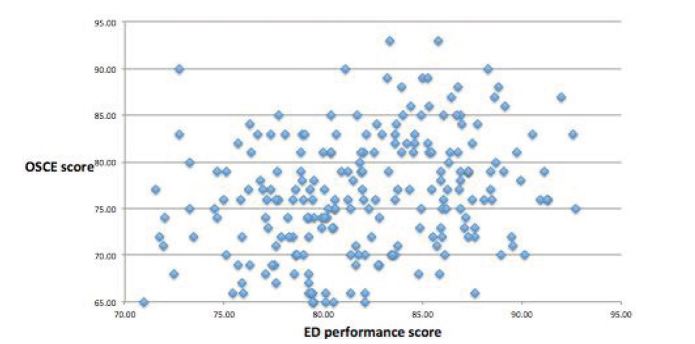
OSCE and ED performance score correlation. *OSCE*, objective structured clinical examination; *ED*, emergency department

Mean OSCE score was 75.0 (SD =7.8), and mean ED performance score was 81.6 (SD =5.4). A positive correlation was found between OSCE score and ED performance score [r(239) =0.40, p <0.001], indicating a statistically-significant linear relationship between the two (Figure 2).

**Figure 3:**
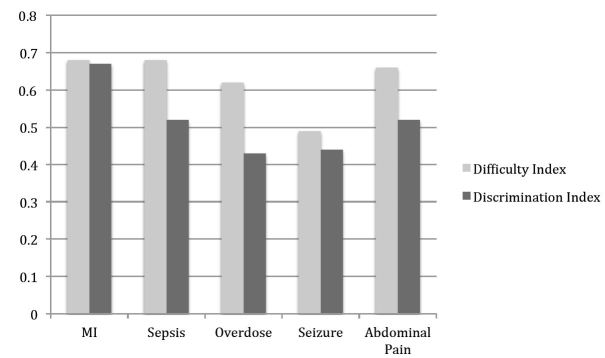
Difficulty and discrimination indices of individual OSCE cases. *OSCE*, objective structured clinical examination; *MI*, myocardial infarction

Of the 34 evaluation items within the five-station OSCE, the mean index of difficulty was 63.0 (SD =23.0) and the mean index of discrimination was 0.52 (SD =0.21). Mean indices of individual cases are demonstrated in Figure 3.

## DISCUSSION

In our EM clerkship, students’ OSCE scores showed a positive and statistically significant correlation with their clinical scores. Based on the computed Pearson’s coefficient the strength of the correlation is moderate. Comparison of difficulty and discrimination indices to best-practice standards show that the majority of our testing items demonstrate ideal characteristics and validates the internal structure of our OSCE evaluation instrument. With regard to difficulty index, 24 (70%) of the total testing items are in the most recommended level I (mid range) and level II (easy) classes, with the remainder in levels III (difficult) and venous line (IV) (extremely difficult or easy), acceptable if used sparingly and in relation to key material.^10^ With regard to discrimination index, 26 (76%) demonstrate “very good” discrimination between high and low performers with an additional five (15%) items demonstrating “reasonably good” discrimination. The remaining three (9%) are marginal or poor and should be revised or eliminated.^11^

A useful model of validity-determination for OSCEs was provided in a 2003 paper by Downing in which he discussed five sources of validity evidence, for each listing examples pertinent to SP-based assessment.^12^ These areas (and SP-relevant examples) include content (selection of cases), response process (evaluation methodology and data integrity), internal structure (test item analysis), relationship to other variables (performance correlation) and consequence (use of method in high-stakes evaluation). The current use of OSCEs as part of the U.S. medical licensure process provides evidence of its consequence validity, and we believe that the deliberate design and implementation of our OSCE program provides evidence of its content and response process validity. Our cases were selected by content-experts and aligned with a national curriculum guideline for EM clerkships. Exacting specifications for patient portrayal were developed, and comprehensive actor training was provided by professional SP educators. Quality control measures were put into place to maximize evaluator accuracy and data integrity.

In this study we have demonstrated the remaining two sources of validity discussed by Downing: internal structure and relationship to other variables. Performance on our OSCE correlates with performance in what is arguably the most common and well-established evaluation method of clinical EM skills, and item-analysis of the OSCE demonstrates characteristics aligned with best-practice testing standards. These data, along with the above-mentioned OSCE characteristics, provide valuable validity evidence for use of an OSCE as an assessment tool for EM clinical skills.

While this study was conducted on undergraduate medical education level, we believe our results are readily generalizable to post-graduate EM education as well. In a clinical environment in which it is difficult to provide standardized and reproducible experiences, the OSCE is a valuable tool that clerkship and program directors can use to asses specific skills in multiple groups of learners. As accrediting and licensing bodies rightfully demand more formal evidence of the acquisition of clinical skills, the need and role for objective, standardized, reproducible and valid assessment such as the OSCE will only increase.

## LIMITATIONS

There are a number of study limitations that may have affected our results. We put significant effort into standardizing the evaluation process during the OSCE. Formal evaluator training was provided and ongoing monitoring was conducted. To promote accuracy of evaluation, we anchored most testing items to the performance of specific tasks rather that a more global assessment of a competency. However, we did not rigorously assess evaluator accuracy nor did we study the inter-rater reliability of the evaluation instrument. This was primarily due to manpower and other practical limitations, although future studies could use video review by multiple evaluators to ensure more accurate performance assessment.

Secondly, our demonstrated correlation between OSCE and ED clinical score, while statistically significant, is only moderate. Sub-optimal inter-rater reliability is one potential variable that may have prevented the demonstration of a stronger correlation, though it may be also be due to the fact that the OSCE and ED performance evaluation, while theoretically similar, in fact evaluate independent performance variables. Additionally, ED performance evaluations by faculty, while well-accepted and established assessment methods in EM education, are subject to numerous biases and limitations, and may not represent a true criterion standard in assessment of clinical skills. Future studies could compare OSCE performance with other measures of clinical skills such as direct observation in the clinical setting and high-fidelity simulation encounters.

Finally, our institution has a well-developed OSCE/SP program, which includes dedicated facilities and technical support, as well a professional SP educator and trainer. These resources, which greatly facilitate our EM OSCE program, may not be available at all institutions. A collaborative multi-center study would both increase our sample size and demonstrate reproducibility at multiple sites.

## CONCLUSION

A management-focused OSCE modified to assess clinical skills relevant to the practice of emergency medicine has validity evidence to support its use in undergraduate EM learners.

## Figures and Tables

**Table:** OSCE case description.

Title	Chief complaint or presenting sign/symptom	Patient demographic	Final diagnosis	Critical actions
Sepsis	Altered mental status	Elderly male or female	Septic shock	Oxygen delivery 2 Liter IV fluid bolus Antibiotic therapy
Seizure	Confused after having seizure	College-age male or female	Bacterial meningitis	Fingerstick glucose Lumbar puncture Antibiotic therapy
Overdose	“Took pills”	Varies	Acetaminophen overdose Depression	Activated charcoal Acetaminophen level NAC therapy
Abdominal pain	Abdominal pain and vaginal bleeding	Young female, 6 weeks pregnant	Missed abortion Intimate partner violence	Ultrasound Communication of bad news IPV Detection & Counseling
MI	Indigestion	Middle-age male	ST elevation MI Ventricular tachycardia	EKG “Cath lab activation” Synchronized cardioversion

*OSCE*, objective structured clinical examination; *IV*, intravenous; *NAC,* N-acetylcysteine; *IPV*, intimate partner violence; *MI*, myocardial infarction; *EKG*, electrocardiogram
